# Temperature Changes (Δ*T*) in Correlation with Number of Implant Osteotomy Preparations in Human Cadaver Tibiae, Comparing Osseodensification (OD) Burs in Clockwise (CW) versus Counterclockwise (CCW) Mode

**DOI:** 10.3390/jfb15080237

**Published:** 2024-08-22

**Authors:** Nikolaos Soldatos, Amanda Heydari, LeRoy Horton, Shayda Sarrami, Luke Nordlie, Dongseok Choi, Robin Weltman

**Affiliations:** 1 Department of Regenerative and Reconstructive Sciences, School of Dentistry, Oregon Health Science University (OHSU), 2730 SW Moody Ave., Portland, OR 97201, USA; heydaria@ohsu.edu (A.H.); hortonle@ohsu.edu (L.H.); shaydasarrami@yahoo.com (S.S.); 2 Department of Oral Rehabilitation and Biosciences, School of Dentistry, Oregon Health Science University (OHSU), 2730 SW Moody Ave., Portland, OR 97201, USA; nordliel@ohsu.edu; 3 OHSU-PSU School of Public Health, Oregon Health & Science University, 1810 SW 5th Ave, Portland, OR 97201, USA; choid@ohsu.edu; 4 Department of Clinical Sciences, School of Dental Medicine, University of Nevada Las Vegas (UNLV), 1001 Shadow Ln., Las Vegas, NV 89106, USA; robin.weltman@unlv.edu

**Keywords:** temperature changes, clockwise mode, counterclockwise mode, osseodensification burs, cortical bone, cancellous bone, human cadaver tibiae, chatter, dental implants

## Abstract

(1) Background: OD burs are used in two different modes: (i) CW and (ii) CCW. The purpose of the study was to evaluate the Δ*T* during the preparation of implant osteotomies in a four-way interaction. (2) Methods: Three hundred and sixty osteotomies were prepared at 12 mm depth in human cadaver tibiae. The Δ*T* values were calculated similarly to the method used in two previous studies carried out by our group. Four different variables were evaluated for their effect on Δ*T*. (3) Results: A four-way interaction was observed in the CCW mode, allowing for 1000 RPM to have the least effect in both modes. However, in the CCW mode the use of 3.0 and 4.0 burs after 23 osteotomies showed a statistically significant increase in Δ*T,* and significant chatter, compared to the CW mode. In the CCW mode, the Δ*T* was increased significantly as the diameter of the burs increased in 800 and 1200 RPM. (4) Conclusions: The synergistic effect of drills’ diameter, CCW mode, 800 and 1200 RPM, and bur usage (over 23 times) had a significant effect on Δ*T*, which exceeded 47 °C. One thousand (1000) RPM had the least effect in both modes. The 3.0 and 4.0 burs in the CCW mode drastically increased the temperature and produced significant chatter.

## 1. Introduction

The initial biomechanical primary stability of dental implants is of paramount importance for osseointegration and long-term success [1]. Factors considered to affect primary stability are the implant thread type and surface, the bone mineral density and type, and the surgical protocol [2,3,4,5,6]. Traditional surgical techniques for osteotomy preparation utilize undersized drilling protocols which provide increased bone-to-implant contact, yielding higher insertion torque values [7].

The concept of OD was introduced with the aim of creating a layer of compacted autogenous bone along the surface of an implant osteotomy, increasing the primary implant stability, bone mineral density, and the percentage of bone-to-implant contact [8]. The OD protocol utilizes specially designed burs (Densah^®^ burs) with chiseled cutting edges and tapered shanks, to develop the length of the osteotomy and a tapered shaft with non-cutting edges to compact the bone laterally and progressively increase the diameter of the osteotomy. CCW rotation along with copious irrigation allows for hydraulic compression along the periphery of the osteotomy [8]. Histologic analyses of osteotomy sites created with the use of these burs confirm the circumferential compaction of alveolar bone and autographing through the appearance of new bone growth on bone chips embedded at the periphery of the osteotomies [9]. Other protocols in implant dentistry where the OD burs can be used in a CCW mode are ridge expansion, the placement of immediate implants, the placement of zygoma implants, and molar septum expansion with or without the placement of an immediate implant. The versatility of the OD burs extends to use in a CW mode, where traditional implant osteotomies can be prepared similarly to conventional implant drills. Interestingly, studies comparing bone mineral density, insertion and removal torque values, and percentage of bone-to-implant contact between the CCW and CW modes with OD burs or specific drills and drilling protocols in implant dentistry showed that both modes using OD burs achieve statistically superior values in all comparisons compared to conventional drilling protocols [9,10,11].

Peri-implant bone loss during the initial healing period and while the implant is still submerged is linked to surgical trauma during the preparation of the osteotomy or implant placement [12,13,14]. Factors that may lead to the bone overheating are bone density, drilling sequence, the design of the drills, the use of external or internal irrigation, the pressure applied to the handpiece from the operator, and the number of times the drills that were used [15,16,17,18,19,20,21,22,23,24,25].

In 2022, the independent and synergistic effect of drill design, diameter, and specific RPM in significantly raising the temperature during CW osteotomy preparations with straight drills and OD burs was reported by our group [26]. The greatest temperature changes occurred early in the osteotomy preparation. As the OD bur diameter increased, the ΔΤ value decreased. The initial pilot bur in the OD group produced the largest temperature changes of 4 °C, 5 °C, and 6 °C at 1000, 1200, 800 RPM, respectively. For both the conventional drills and the OD burs, one thousand (1000) RPM had a lower effect on ΔΤ compared to 800 and 1200 RPM. The magnitude of the increases in the temperature readings throughout the osteotomy process did not exceed the critical threshold of 47 °C, which could lead to necrosis of the alveolar bone and impairment of bone healing which could compromise the implant osseointegration [26].

Bhargava et al. [27] compared temperature changes and bone architecture following osteotomy preparations with osteotomes, piezoelectric technology, the use of OD burs in the CCW rotation, and conventional drilling. The drilling protocols were carried out at 1100 RPM. The temperature changes were calculated by subtracting the temperature readings prior to initiating the drilling sequences from the temperature readings at the completion of the drilling sequences. The piezoelectric and OD processes increased the temperatures within the cortical bone core of the porcine rib specimens by 5 °C and 1 °C, respectively, with negligible changes occurring due to the other two techniques. Within the cancellous bone levels, the piezoelectric technique registered slightly smaller temperature increases, while the OD and other techniques registered minimum to no changes in temperature [27].

The purpose of the present study was to assess the effect on Δ*T* on a four-way interaction between the following variables, during the preparation of 60 implant osteotomies per group: (i) CW compared to CCW mode, (ii) three different RPMs (800, 1000, and 1200), (iii) drill diameter, and (iv) the use of OD burs. The null hypotheses were (i) the CW and CCW use of OD burs will produce the same amount of heat generation in every RPM, and (ii) using the burs 60 times per group will result in the same amount of heat generation in every RPM and mode.

## 2. Materials and Methods

No institutional review board (IRB) approval was required for the completion of the present human cadaver study. The OHSU IRB reviews research that involves human subjects. Cadavers are not considered human subjects. The cadavers were de-identified and the four examiners (N.S., A.H., S.S., and L.H.) could not identify the subjects. The cadavers were donated for clinical and research purposes to the cadaver donation and VirtuOHSU^®^ simulation and surgical training center at the Richard T. Jones Hall for Basic Medical Sciences, in Portland, Oregon. The relatives signed all the appropriate informed consent forms, and the cadavers were examined through blood testing to ensure the safety of the present study. The methodology was reviewed and approved by an independent statistician (D.C.). Three unembalmed cadavers (two male and one female) were used in the present study. All three cadavers were between 77 and 79 years old and were deceased between December 2022 and January 2023. The causes of death were pulmonary fibrosis, respiratory failure, and ovarian cancer, respectively. All three cadavers were freshly frozen and stored at 5 °F. The cadavers were placed in a cooler at 42 °F to thaw for 3 days prior to the beginning of the present study. The study took place on February 2023. 

Human mandibular and tibial bones, although having different origins, have similar mechanical properties such as their compressive strength and modulus of elasticity [28]. An innovative translational model using human cadaver tibiae under the plateau was used in the present study. The model was previously developed and validated by one of the authors (N.S.) and has already been used in two previous studies [26,29]. Six six-inch-long unembalmed tibial sections were harvested bilaterally from all three cadavers and placed in water baths, similar to the method used the previous studies carried out by our group [26,29].

Three temperature-regulated digital laboratory water baths (IVYX Scientific, Seattle, WA, USA), filled with sterile saline, were maintained at a range of 95.2 °F to 99.6 °F (35.1–37.5 °C) to simulate the normal physiologic human body temperature. The room temperature was kept at a constant 68 ± 1 °F (20 °C).

**Figure 1:**
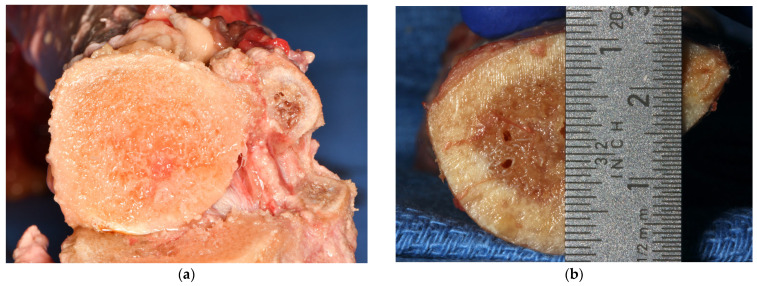
(**a**,**b**) Two tibial sections, harvested right under the plateau, with 5 mm cortical and 7 mm cancellous bone.

**Figure 2:**
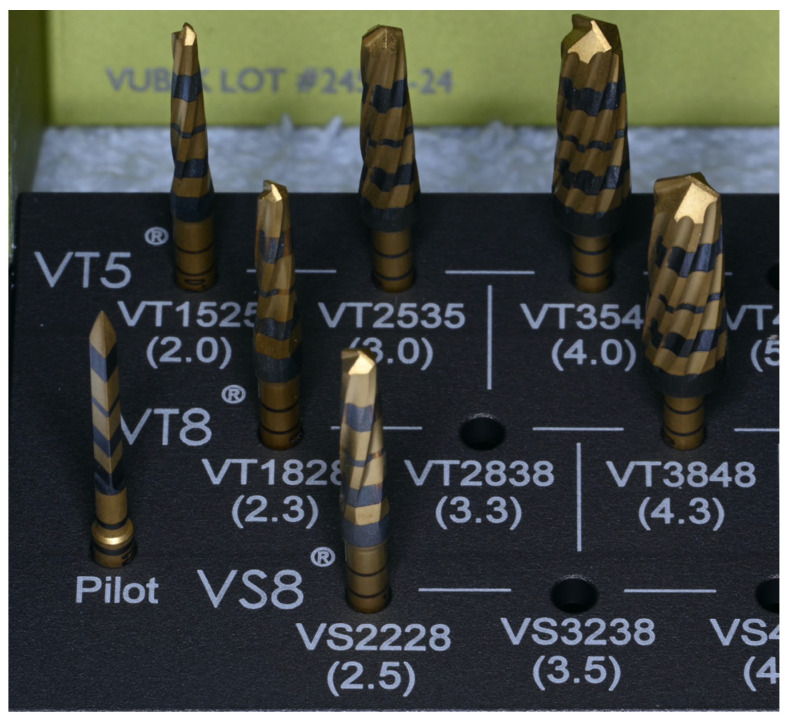
The bur sequence protocol (1.6, 2.0, 2.3, 2.5, 3.0, 4.0, and 4.3) used for the preparation of the osteotomies in every group, according to manufacturer’s recommendations of a ∅5.0 mm × 12 mm bone level implant.

The study design allowed for the preparation of 60 osteotomies per group, using OD burs. The length of each osteotomy was 12 mm (5 mm cortical and 7 mm cancellous bone). The temperature of the tibiae and the osteotomies were recorded with a K-type thermocouple (Fisher Scientific^®^, Hampton, NH, USA 15-078-187, range −58 to 2000 °F, resolution 0.1°/1°, sampling rate 2.5 times per second), with an ultra-fast response naked bead probe (maximum range 260 °C), respectively [26,29]. The thermocouple was programmed to measure the maximum temperature during the preparation of the osteotomies. Figure 1a,b shows two tibial sections right after harvesting. These tibiae are an accurate representation of the six tibial sections used in the study. The OD burs used for the preparation of the osteotomies were donated by Versah^®^ LLC (Jackson, MI, USA). The bur sequence suggested by the manufacturer for the placement of a ∅5.0 mm × 12 mm bone level implant (burs 1.6, 2.0, 2.3, 2.5, 3.0, 4.0, and 4.3) was followed for all groups (Figure 2). 

Six groups were formed: three for CW (800-1000-1200 RPM) and three for CCW (800-1000-1200 RPM). For each group, a new set of burs was used. Four examiners (N.S., A.H., S.S., and L.H.) were working on the preparation of the osteotomies; therefore, they were previously calibrated to avoid any inconsistency during the data collection. 

Calibration: Calibration of the examiners was completed using a protocol previously described in two studies of our group [26,29]. 

**Figure 3:**
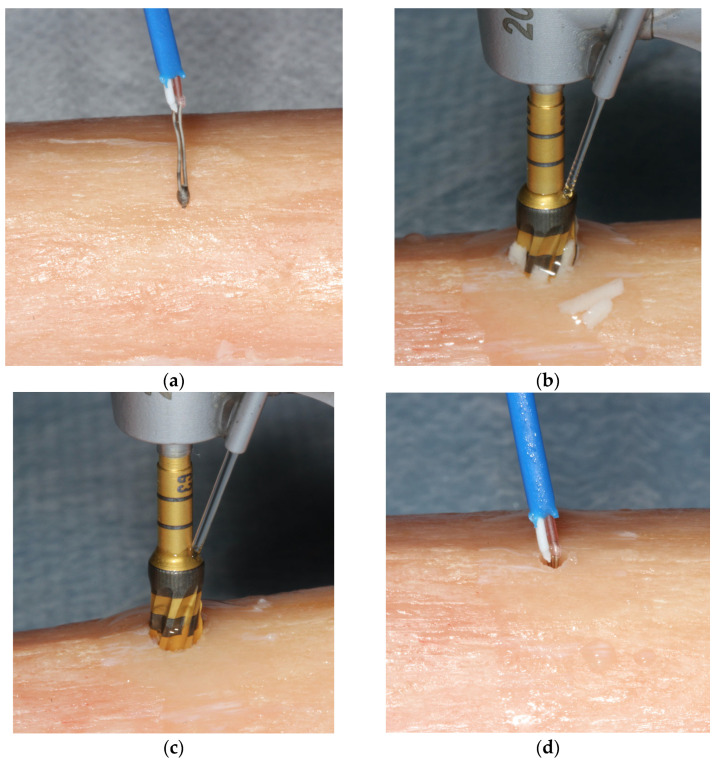
(**a**) The thermocouple probe recording a baseline temperature at the osseous surface. (**b**) CW implant osteotomy preparation with external irrigation. Note the characteristic bone chips trapped during drilling at the flutes of the bur. (**c**) CCW implant osteotomy preparation with external irrigation. The characteristic bone chips trapped at the flutes of the bur, seen previously at CW preparation, are absent since the bone is densified over the walls of the osteotomy. (**d**) The thermocouple probe recording the maximum temperature over the walls of the osteotomy.

The examiners (N.S., A.H., S.S., and L.H.) rotated between the CW and CCW groups every 5 osteotomies. The tibial sections were removed from the water bath and placed on a countertop. A baseline temperature measurement was recorded on the osseous surface prior to preparation. Implant osteotomy preparations were performed either in CW or CCW mode. Then, the probe was inserted into the osteotomies’ walls immediately following the osteotomy preparation with each consecutive drill and the temperature was measured and recorded. (Figure 3a–d).

Δ*T* was calculated by subtracting the baseline temperature from the maximum temperature recorded immediately after drilling for each drill diameter (∆*T* = *T*max − *T*baseline). After 2–3 osteotomies, the tibial sections were returned to the water baths to maintain the temperature as close to the temperature of the human body within the bounds of study protocol. To allow the dispersed heat to dissipate before another osteotomy was performed, consecutive osteotomies were performed on opposite ends of the tibial sections. Sixty (60) osteotomies were prepared, at 12 mm depth per group, to allow for a total of 360 osteotomies. All values were recorded on an Excel^®^ (Redmond, WA, USA) spreadsheet. 

Statistical analyses: For the statistical analyses, the R statistical software was used (R Core Team 2021, R Foundation for Statistical Computing, Vienna, Austria) [30]. The variables [(i) mode; CW compared to CCW, (ii) RPM, (iii) drill diameter, and (iv) usage number of OD burs] were evaluated both for their individual and for their synergistic effects on ∆T with the use of one-, two-, three-, and four-way interactions between the variables. 

## 3. Results

**Figure 4:**
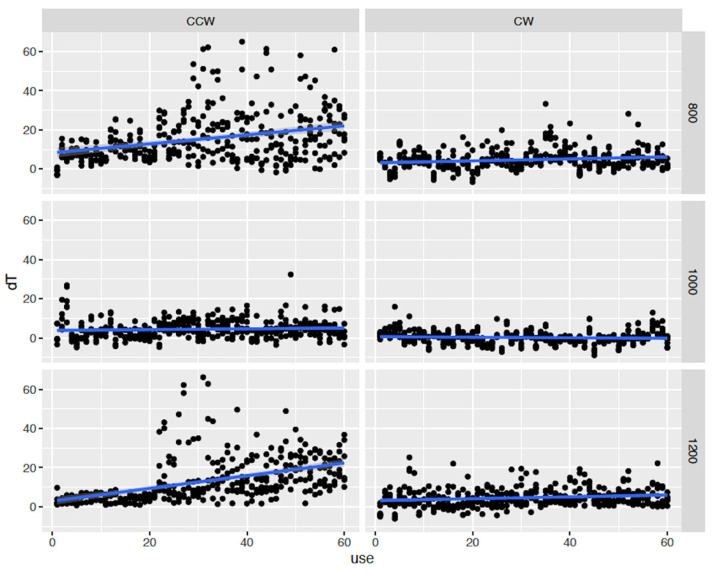
View of both modes, Δ*Τ*, three different RPMs, and number of osteotomies. In the CCW mode, the Δ*T* was significantly affected and was increased at 800 and 1200 RPM. In both modes, one thousand RPM had no significant effect in Δ*T*.

**Figure 5:**
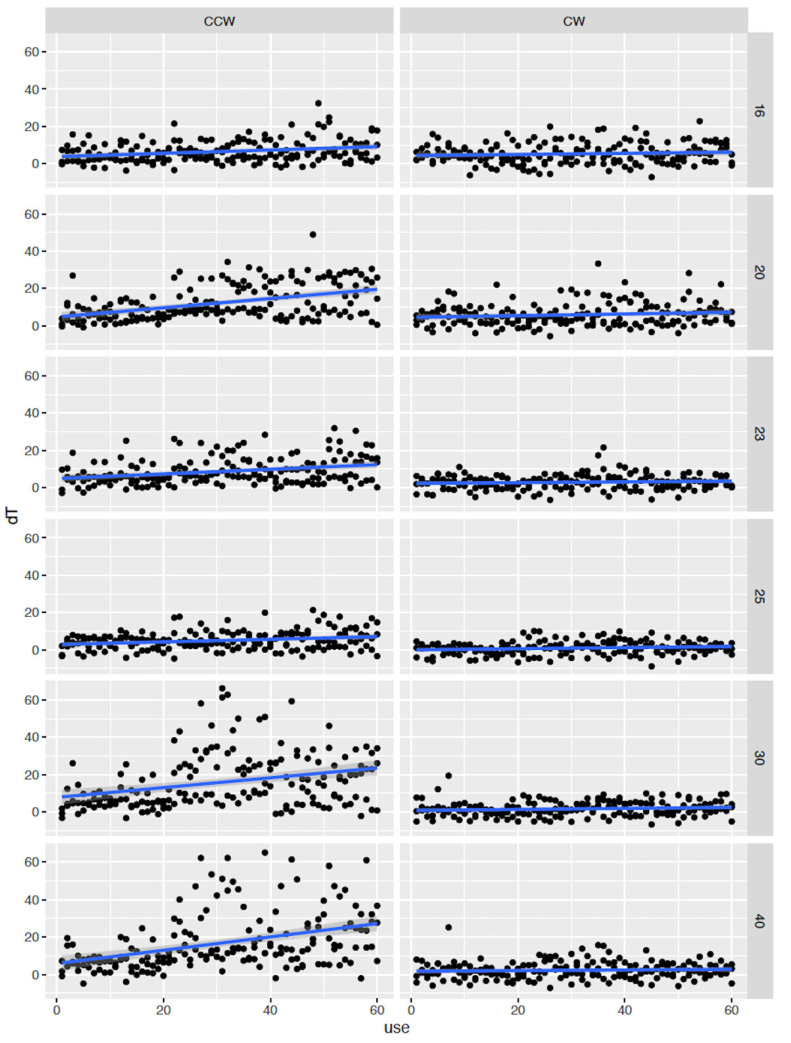
View of the CW and CCW modes along with the number of the osteotomies and the burs used, based on the manufacturer’s protocol. The 2.0 bur began to chatter after 32 osteotomies at 1200 RPM. Both the 3.0- and 4.0 mm burs began to chatter after 23 osteotomies allowing for consistent high Δ*T* and high overall temperature which exceeded the critical threshold of 47 °C.

Table 1 and Figure 4 and Figure 5 demonstrate the results of the present study. Table 1 shows a four-way interaction between the variables and Δ*T.* All the variables independently and synergistically had a significant impact on Δ*T.* There was a statistically significant difference between the CW and CCW modes. 

Figure 4 illustrates both modes, all three RPMs, and the use of the burs. In the CCW mode and at 800 and 1200 RPM, the Δ*T* was significantly affected and raised over the critical threshold of 47 °C. The use of burs at 1000 RPM had the least effect on Δ*T* in both modes. The first chatter was evident after 23 and 26 osteotomies at 1200 and 800 RPM, respectively, in the CCW mode, as a type of vibration during the drilling process led to an inaccurate drilling depth, compromised the stability of the implant site, and damaged the surrounding bone tissue. The chatter allowed the Δ*T* to go as high as 68 °C, and the overall temperature was 98.1 °C. No chatter was noted in the CW mode. 

Figure 5 shows that all burs, except 1.6 bur, had chatter at the CCW mode which significantly affected Δ*T*. The 2.0 bur began to chatter after 32 osteotomies at 1200 RPM. Both 3.0- and 4.0 mm burs began to chatter after 23 osteotomies. After the evidence of chatter, the burs had consistently high Δ*T* values and a high overall temperature for up to 60 osteotomies, exceeding the critical threshold of 47 °C. Two additionally significant findings were (i) the suspension of the 4.3 mm bur from the study after 35 osteotomies in both modes, since it was impossible to latch it into the handpiece due to a bent shaft and a broken latch, and (ii) the spring-back effect in the CCW mode, which was more noticeable at 1000 RPM. The spring-back effect did not allow the same bur to be placed back into to the osteotomy after the completion of the preparation. 

**Figure 6:**
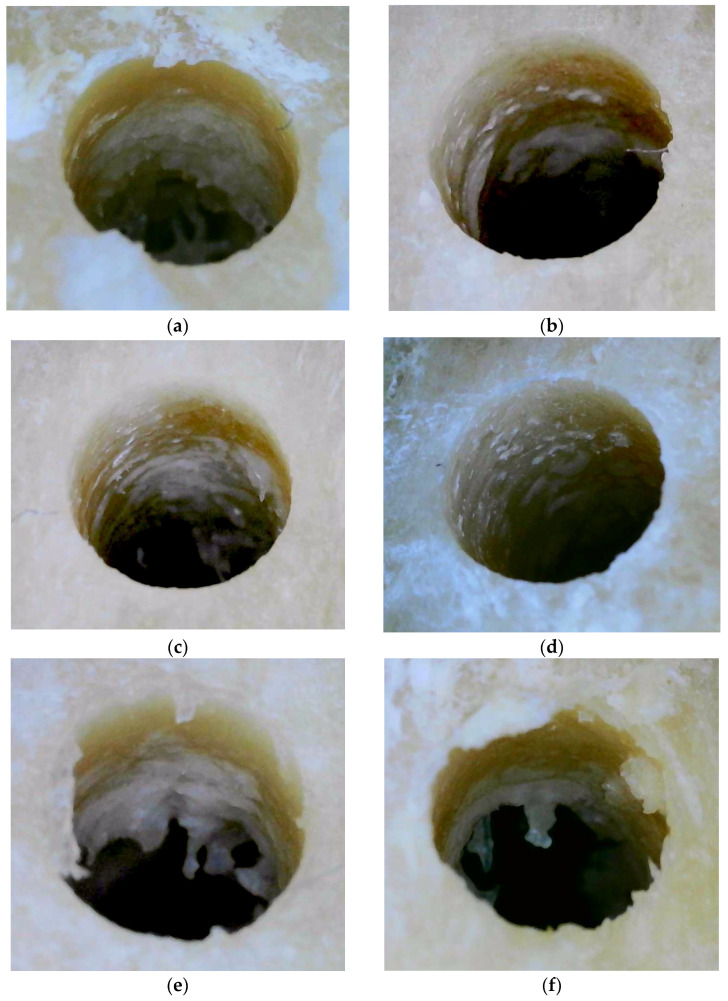
View of stereoscopy imaging of CW (**a**) and CCW (**b**) modes at 800 RPM. View of stereoscopy imaging of CW (**c**) and CCW (**d**) modes at 1000 RPM. Note the optimum OD in the CCW mode compared to 800 and 1200 RPM. View of stereoscopy imaging of CW (**e**) and CCW (**f**) modes at 1200 RPM.

Stereoscopy imaging: Stereoscopy imaging was performed similarly to Soldatos et al. 2022 [26]. A separate tibial section was used for the preparation of six osteotomies with the use of OD burs: (i) CW-CCW at 800 RPM, (ii) CW-CCW at 1000 RPM, and (iii) CW-CCW at 1200 RPM (Figure 6a–f). The tibial section with the six osteotomies was submerged in sterile water before stereomicroscopic images were taken. The submerged sections were then placed under the objective lens of a Nikon^®^ Stereomicroscope SMZ 800 (Melville, NY, USA) and images were taken at 40× magnification. The specimen in the 1000 RPM CCW mode showed the most densified bone compared to 800 and 1200 RPM. The CW mode at all three different RPMs, showed similar irregularities over the osteotomy walls (except for 1000 RPM), suggesting uncondensed bone. The specimen with the 1000 RPM CW mode was in accordance with the results of the present study, showing a mixture of condensed and uncondensed bone. 

## 4. Discussion

The present study compared multiple variables at one time, similar to what would be addressed in a clinical environment. The purpose was to assess the effect on Δ*T* of a four-way interaction between the following variables during the preparation of 60 implant osteotomies: (i) CW compared to CCW mode, (ii) three different RPM (800, 1000 and 1200), (iii) drill diameter, and (iv) the usage of OD burs. Both null hypotheses were rejected since there was a statistically significant difference between the CW and CCW modes at all RPM (except 1000 RPM), the drill size had a significant effect, and using the burs more than 23 times significantly elevated the temperature (over 47 °C) which subsequently significantly affected the Δ*T.* One thousand (1000) RPM had the least effect on Δ*T* in both modes, confirming our previous findings [26]. The critical temperature point, which can compromise the bone around an implant, was described as being >47 °C for one minute, since it significantly reduced the bone formation around implants [31,32]. To measure the temperature, a K-type thermocouple measuring unit was utilized in the present study due to its higher accuracy in a liquid medium compared to infrared thermography [33,34,35]. The measurement of the temperature was performed directly into the osteotomy since it was reported that there is a 1.5 °C difference in temperature between distances of 0.3 mm and 0.5 mm from the osteotomy site [24]. The CW results of the present study are in accordance with Soldatos et al. 2022 [26], where the same burs were used to prepare 40 osteotomies in the same human cadaver model.

The present study, and previous studies from the same group, used a high translational human cadaver model using the tibial bone under the plateau; however, several studies have been performed using bone substitutes. The model in the present study had 5 mm of cortical and 7 mm of cancellous bone. Cortical and cancellous bone show different healing responses and heat dispersal during osteotomy preparations, since there is anatomical variance between them [36]. In addition, the porosity of the alveolar bone differs between cortical (3.5%) and cancellous bone (79.3%) [37]. The most common bone substitutes are solid rigid polyurethane foam bone blocks, which are used as an alternative test medium to human bone (Sawbones^®^, Vashon Island, WA, USA). This type of bone was used for calibration in the present study and the previous study by our group [38]. Romeo et al. [11], on artificial bone substitutes, focused on the use of OD and conventional burs in CW and CCW modes, and their relationship to implant stability measurements obtained by insertion torque and resonance frequency analysis. They found that the OD burs in the CCW mode allowed for significantly higher insertion and removal torques for bone-level tapered implants. However, 600 RPM was used for all burs, which is not recommended by the manufacturers of both the OD and the conventional burs. In addition, they noticed that the final diameter of the osteotomy created with the OD burs in the CCW mode was narrower than the other drilling modalities due to the spring-back effect of the cancellous bone after drilling, a finding that was noticed in the present study as well [11]. The spring-back effect was described by Kold et al. [39] as a response of compacted bone which reduces the size of the osteotomy [39]. Huwais and Meyer [8] reported, in a porcine tibial model, that the spring back effect is due to the viscoelastic portion of the deformation, causing a 91% reduction in the OD osteotomy size when it was left empty during microcomputed tomography [8]. 

Many studies have used different models, different temperature measuring devices, different RPM, different drill designs, and different locations for temperature capture to address the temperature changes during implant osteotomy preparations [26,29,36,38,40,41,42,43,44,45,46,47,48]. All these studies have used the drills in CW mode. Trisi et al. [36] found that, in an iliac crest sheep model, a temperature of 60° for 1 min significantly reduced the bone-to-implant contact [36]. Dos Santos et al. [40], in a rabbit tibial model, found that a guided drilling protocol produced higher temperature than the conventional. The temperature was increased with the number of times the drills were used; an opposite finding to that for the CW mode group in the present study [40]. Like Dos Santos et al. [40], Barrak et al. [41] evaluated the intraosseous temperature during guided and free-hand osteotomy preparations. The model and the protocol were different as they used bovine ribs at 800, 1200, 1500, and 2000 RPM. The guided group significantly elevated the temperature over the critical threshold of 47°C, with the metal sleeve of the guide, the higher RPM, the sterilization protocol of the drills and the number of the osteotomies performed with the same drills being significant contributing factors to the elevation of the temperature [41]. In 1972, Matthews and Hirsch [42] reported temperatures more than 100 °C when they drilled human cortical bone without irrigation under laboratory conditions. In addition, worn drills and the force applied to the drill were more important factors for increasing the temperature than the drilling speed [45]. Benington et al. [43], in a bovine mandibular model using the Branemark technique for implant placement, reported temperatures as high as 130.1 °C when three different drills were used [46]. Three different studies from Misir et al. [44], Jochum et al. [24], Oliveira et al. [45], and Allsobrook et al. [46] described a non-significant elevation of the temperature after the use of drills 45, 50, 50 and 40 times, respectively [24,44,45,46]. In the present study, after 60 osteotomies in the CW group, there was no significant elevation of the temperature. Chacon et al. [15] measured the temperature generated by straight design drills using sequential drilling up to 4–4.2 mm diameters. Only the drill design without a relief angle yielded a bone temperature above 47 °C [15]. Scarano et al. [47] found that the triple twist cylinder drills generated more heat than the quadruple twist conical drills on a cortical bovine bone model [47]. Finally, our group discovered a three-way interaction between ΔΤ and drill design, drill diameter, and RPM. A clear pattern appeared for the OD burs at all RPMs after they were used 40 times in CW mode [26]. 

The chatter reported in the present study after 23 times in the CCW mode can negatively impact the success of the implant placement procedure by causing irregular bone preparation and affecting the overall integration and longevity of the implant. Surgical providers aim to minimize chatter by replacing the drills according to manufacturer’s recommendations and using appropriate drill techniques and specific RPMs and equipment to ensure precise and controlled drilling during implant surgery [48]. 

The examination of the specimens through stereoscopy imaging proved the OD of the CCW mode (especially in 1000 RPM), as was previously reported [26]. Temperature changes during dental implant osteotomy preparations can have significant effects on the success of the implant surgical procedure. Optimization through proper selection of drilling parameters and use of irrigation are crucial to minimize Δ*T* and reduce the risk of complications. To the best of the authors’ knowledge, this is the first human cadaver study measuring Δ*T* by comparing the two different modes of the OD burs for the preparation of dental implant osteotomies. The authors identified two limitations of the study; (i) the in vitro nature on a fresh human cadaver model, which does not account for the blood and salivary flow of a patient, and the in vivo intraosseous bone temperatures, and (ii) even though the thermocouple is a very precise method of temperature measurement, real-time temperature readings through the handpiece could offer even more accurate results. 

## 5. Conclusions

The synergistic effect of the CCW mode, drills’ diameter, 800 and 1200 RPM, and use of burs over 23 times had a significant effect on Δ*T* in human cadaver tibiae which exceeded the critical threshold of 47 °C. Significant chatter was produced at almost every bur in the CCW mode after they had been used over 23 times. One thousand (1000) RPM had the least effect in both modes. 

## Figures and Tables

**Table 1:** ANOVA table shows a four-way interaction between the variables and Δ*T* [Abbreviations used in the table: U (Use), M (Mode), DW (Drill Width)].

	DF	Sum Sq	Mean Sq	F Value	Pr (>F)
U	1	7808	7808	209.270	<2 × 10^−16^ ***
M	1	31757	31757	851.198	<2 × 10^−16^ ***
DW	5	11741	2348	62.942	<2 × 10^−16^ ***
RPM	2	24320	12160	325.923	<2 × 10^−16^ ***
U:M	1	4321	4321	115.826	<2 × 10^−16^ ***
U:DW	5	1732	346	9.285	9.30 × 10^−9^ ***
M:DW	5	13320	2664	71.404	<2 × 10^−16^ ***
U:RPM	2	3889	1944	52.119	<2 × 10^−16^ ***
M:RPM	2	3650	1825	48.921	<2 × 10^−16^ ***
DW:RPM	10	4670	467	12.518	<2 × 10^−16^ ***
U:M:DW	5	1819	364	9.750	3.20 × 10^−9^ ***
U:M:RPM	2	1614	807	21.631	5.03 × 10^−10^ ***
U:DW:RPM	10	1943	194	5.208	1.39 × 10^−7^ ***
M:DW:RPM	10	3720	372	9.970	<2 × 10^−16^ ***
U:M:DW:RPM	10	2083	208	5.584	2.89 × 10^−8^ ***

*** Indicates statistical significance < 0.0001.

## Data Availability

The original contributions presented in the study are included in the article, further inquiries can be directed to the corresponding author.
